# Increased risk of breast cancer-specific mortality among cancer survivors who developed breast cancer as a second malignancy

**DOI:** 10.1186/s12885-021-08132-9

**Published:** 2021-05-03

**Authors:** Chengshi Wang, Kejia Hu, Lei Deng, Wei He, Fang Fang, Rulla M. Tamimi, Donghao Lu

**Affiliations:** 1 Laboratory of Molecular Diagnosis of Cancer, and Department of Medical Oncology, Clinical Research Center for Breast Diseases, West China Hospital, Sichuan University, Chengdu, Sichuan PR China; 2 Institute of Environmental Medicine, Karolinska Institutet, Stockholm, Sweden; 3 Department of Medicine, Roswell Park Cancer Institute, Buffalo, NY USA; 4 Department of Medical Epidemiology and Biostatistics, Karolinska Institutet, Stockholm, Sweden; 5 Department of Epidemiology, Harvard T.H. Chan School of Public Health, Boston, MA USA; 6 Department of Population Health Sciences, Weill Cornell Medicine, New York, NY USA; 7 West China Biomedical Big Data Center, West China Hospital, Sichuan University, 37 Guo Xue Xiang, Chengdu, Sichuan 610041 PR China

**Keywords:** Second primary breast cancer, Breast cancer-specific mortality, Prognosis, Survival

## Abstract

**Background:**

Cancer survivors who develop breast cancer as a second malignancy (BCa-2) are common. Yet, little is known about the prognosis of BCa-2 compared to first primary breast cancer (BCa-1).

**Methods:**

Using the Surveillance, Epidemiology, and End Results database, we conducted a population-based cohort study including 883,881 patients with BCa-1 and 36,313 patients with BCa-2 during 1990–2015. Compared with patients with BCa-1, we calculated hazard ratios (HRs) of breast cancer-specific mortality among patients with BCa-2, using multivariable Cox regression.

**Results:**

During the follow-up (median 5.5 years), 114,964 and 3829 breast cancer-specific deaths were identified among BCa-1 and BCa-2 patients, respectively. Patients with BCa-2 had more favorable tumor characteristics and received less intensive treatment e.g., surgery and chemo−/radio-therapy, compared to patients with BCa-1. When adjusting for demographic factors, patients with BCa-2 were at similar risk of breast cancer-specific mortality (HR 1.00, 95% CI 0.97–1.03) compared to patients with BCa-1. However, when additionally controlling for tumor characteristics and treatment modes, BCa-2 patients were at an increased risk of breast cancer-specific mortality (HR 1.11, 95% CI 1.08–1.15). The risk elevation was particularly greater when the first malignancy was lung, bladder, ovarian or blood malignancy (HRs 1.16–1.85), or when the first malignancy was treated with chemotherapy and radiotherapy (HR 1.44, 95% CI 1.28–1.63).

**Conclusions:**

Overall, patients with BCa-2 have worse breast cancer-specific survival, compared with their BCa-1 counterparts, although the risk elevation is mild. High-risk subgroups based on first malignancy’s characteristics may be considered for active clinical management.

**Supplementary Information:**

The online version contains supplementary material available at 10.1186/s12885-021-08132-9.

## Background

More people are surviving cancer and with the increased survival comes opportunities for second primaries [1]. Second primary cancer has significantly increased over recent decades, accounting for 8–10% of newly diagnosed cancers in the U.S. and Australia [2, 3]. Breast cancer is the most common type, accounting for almost half of second cancer developed among female cancer survivors in the U.S. [2, 4]. It was recently reported that the incidence of second primary breast cancer has been increased by 600% from 1994 to 2015 in the U.S. [5], although the clinical course has not been studied yet. The vast majority of research attention was devoted to contralateral breast cancer (CBC) [6], however, it only accounts for approximately 3% of primary breast cancers as a second malignancy [3]. It is well-recognized that the prognosis of CBC is inferior to first primary breast cancer [7], whereas little is known about primary breast cancer developed among survivors of non-mammary malignancy (referred to as BCa-2 below).

Emerging evidence suggests that BCa-2 may have different pathogenesis or biological behaviors compared with BCa-1. This is partly attributable to intensive cancer treatment for the first malignancy (e.g., radiotherapy [8] and chemotherapy [1]), as well as certain lifestyle (e.g. smoking [9, 10]) and genetic factors [11, 12] that predisposed the individual to both first and second malignancies. Compared with the general population, high-dose chest radiation or exposure to alkylator or anthracycline was associated with drastically increased risk of subsequent breast cancer among childhood cancer survivors [13–16]. Moreover, the tumor characteristics of BCa-2 appear to differ. Compared with BCa-1, BCa-2 after Hodgkin’s lymphoma (HL) were characterized by early stage, hormone receptor negative status, and more likely to be located in external quadrant of breast [15, 17].

To aid the clinical management of an increasing number of BCa-2, it is important to understand whether the prognosis of BCa-2 is different from BCa-1. Milano et al. [18] reported worse breast cancer-specific survival among HL survivors with localized, but not regional or distant, BCa-2. It is however unclear whether BCa-2 as a whole have a worse disease course than BCa-1. Leveraging the population-based cancer cohort from the Surveillance, Epidemiology, and End Results (SEER) database, we aimed to assess the risk of breast cancer-specific mortality among cancer survivors of non-mammary malignancy who developed BCa-2 as a second malignancy, and identify potential high-risk subgroups due to the associations of first malignancy.

## Methods

### Study population

**Fig. 1:**
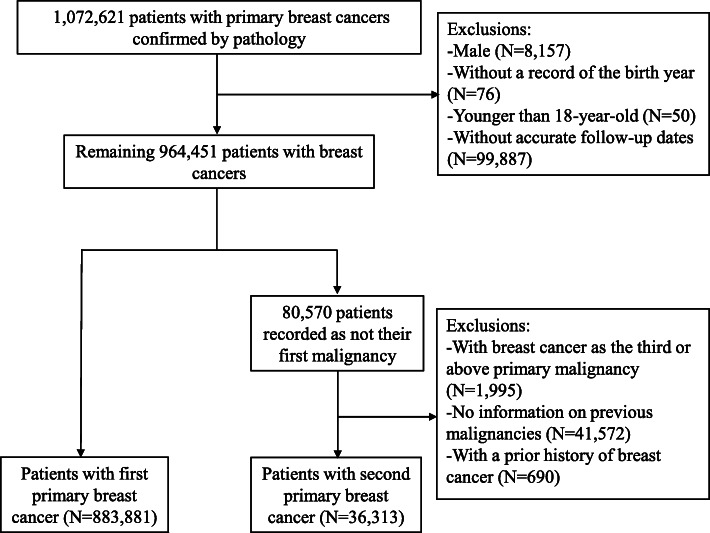
The flowchart

The SEER database contains information on demographic, tumor and clinical characteristics, and follow-up from nine registries (SEER9), in 1973, and expanded to 13 registries (SEER 13), in 1992 and 18 (SEER 18), in 2000 (covering about 28% of the US population) [19]. Considering the data for both the status of estrogen receptor and progesterone receptor was collected from 1990, we conducted a population-based cohort study of patients with primary breast cancer diagnosed between January 1, 1990 and December 31, 2015 in the United States. Similar to our previous study [20], we first identified 1,072,621 patients with pathologically confirmed, primary invasive breast cancer; and then excluded patients who were male (*N* = 8157), without a record of birth year (*N* = 76), or younger than 18 years at diagnosis (*N* = 50). All patients were followed from breast cancer diagnosis until death, occurrence of a subsequent malignancy, or December 31, 2015, whichever occurred first, whereas patients without accurate follow-up (including incomplete dates available, complete dates available but 0 days of survival, or unknown follow-up dates) were excluded (*N* = 99,887). The exclusions were illustrated in Fig. 1.

### Ascertainment of BCa-2

In these breast cancer cases (*N* = 964,451), 80,570 were coded as non-first primary malignancy. Through linkage to the information on previous diagnoses in SEER, we excluded breast cancer cases diagnosed after two primary malignancies of which the clinical course may be very different from BCa-2 (*N* = 1995; and 41,572 cases without any identifiable diagnosis of the first malignancy (the baseline characteristics and mortality rates were summarized in Supporting Information Table S1 and were comparable to the patients with BCa-2 included in the final analysis). BCa-2 patients with a prior history of breast cancer were also excluded (*N* = 690) because of the difficulty to determine deaths from BCa-1 or BCa-2. In the present study, BCa-2 is therefore restricted to primary breast cancers subsequent to a non-mammary malignancy. The ten most common sites of first primary malignancies were colon and rectum, corpus and uterus, blood, skin, lung and bronchus, thyroid, ovary, urinary bladder, kidney and cervix uteri (Supporting Information Table S2). Finally, 36,313 BCa-2 and 883,881 BCa-1 were included for analysis.

### Ascertainment of mortality

Breast cancer-specific and overall mortality were considered as the primary and secondary outcomes, respectively. Patients registered in the SEER program are followed periodically by linking registries through health care institutions and by directly contacting patients. To ensure the maximal follow-up of patients, personal contacts are also periodically implemented for those who are considered lost to follow-up [20, 21].

SEER uses algorithms to determine the single, disease-specific cause of death utilizing information on death certificates, tumor sequence, tumor site, and comorbidities [21, 22]. We identified deaths due to breast cancer using SEER Cause of Death Recode (code: 26000) [22, 23], of which the quality has been validated [24] in BCa-1.

### Demographic, tumor and clinical characteristics

We extracted information on calendar year at diagnosis (1990–1993, 1994–1997, 1998–2001, 2002–2005, 2006–2009, or 2010–2015), age at diagnosis, race (White, Black, Asian, or other), and marital status (non-cohabitation, cohabitation, or unknown). Information on educational level and cost of living was obtained at the county level and classified into low, middle and high groups based on tertiles. We obtained information on tumor stage (localized, regional, distant, or unknown), tumor size (0–2 cm, 2–5 cm, or > 5 cm), histology (ductal, lobular, mixed, or other origins), tumor grade (well, moderately, or poorly differentiated, undifferentiated, or unknown), status of estrogen receptor (ER), progesterone receptor (PR), hormone receptor status (i.e., combining ER and PR status), and human epidermal growth factor receptor 2 (HER2, available from 2010 onward). Molecular subtype (available from 2010 onward) was classified as hormone receptor positive (HR+)/HER2-, HR+/HER2+, hormone receptor negative (HR-)/HER2+, triple negative, or unknown. We also extracted information on treatment, including surgery (mastectomy, lumpectomy, or no/unknown; available from 1998 onward), radiotherapy (yes or no/unknown), and chemotherapy (yes or no/unknown). To better reflect the treatment modes, we further classified treatment into lumpectomy only, mastectomy only, chemo−/radio-therapy, lumpectomy plus chemo−/radio-therapy, mastectomy plus chemo−/radio-therapy, and others.

### Statistical analysis

First, we compared tumor and clinical characteristics between patients with BCa-2 and BCa-1 using logistic regression models with adjustment for demographic characteristics and tumor characteristics (only in the analysis of treatment modes).

Next, we calculated the mortality rates and hazard ratios (HRs) of breast cancer-specific and overall mortality among patients with BCa-2, as compared to patients with BCa-1, using Cox regression where we graphically assessed the assumption in a log-log plot. It is known that childhood cancer survivors are at higher risk of breast cancer due to the higher doses of anthracyclines [25]. To illustrate that the associations were not driven by childhood cancer survivors, an additional analysis was performed by excluding women who were aged ≤20 years at the diagnosis of first primary malignancy. To alleviate the concerns that some deaths due to first malignancy were misclassified as due to BCa-2, we performed a sensitivity analysis by restricting to BCa-2 diagnosed > 10 years after the first malignancy, when the first malignancy was presumably cured.

In these analyses, we adjusted for demographic characteristics as Model A, and additionally controlled for tumor characteristics (Model B) and treatment modes (Model C). Age at diagnosis was used as a continuous variable, while other factors were categorized as shown in Tables 1 and 2. In order to show associations independent of tumor characteristics and treatment modes, we only applied Model C in analyses below.

**Table 1:** Baseline characteristics of women with primary breast cancer as the first (BCa-1) and second (BCa-2) malignancy: a SEER population-based study in US, 1990–2015

	BCa-1		BCa-2	
	N	%	N	%
**Total number**	883,881	–	36,313	–
**Year of diagnosis**				
1990–1993	47,727	5.4	1806	5.0
1994–1997	62,863	7.1	2380	6.6
1998–2001	117,368	13.3	3332	9.2
2002–2005	165,528	18.7	5250	14.5
2006–2009	185,587	21.0	7775	21.4
2010–2015	304,808	34.5	15,770	43.4
**Age at diagnosis (mean ± SD), years**	60.5 ± 14.0		68.9 ± 12.4	
**Race**				
White	708,820	80.2	30,929	85.2
Black	97,049	11.0	3086	8.5
Asian	68,641	7.8	2127	5.9
Other	9371	1.1	171	0.5
**Cohabitation status**				
Non-cohabitation	359,775	40.7	17,217	47.4
Cohabitation	484,233	54.8	17,252	47.5
Unknown	39,873	4.5	1844	5.1
**% of High-school education in the county of residence**				
Lowest tertile	173,646	19.6	6202	17.1
Middle tertile	303,827	34.4	10,779	29.7
Highest tertile	406,408	46.0	19,332	53.2
**Cost of living adjusted median household income in the county of residence**				
Lowest tertile	54,584	6.2	1885	5.2
Middle tertile	116,136	13.1	4648	12.8
Highest tertile	713,161	80.7	29,780	82.0

*Abbreviations*: *N* Number, *SD* Standard deviation

**Table 2:** Associations of tumor characteristics and treatment modes with primary breast cancer as the second malignancy (BCa-2): a SEER population-based study in US, 1990–2015

	**BCa-1**	**BCa-2**	
	**N (%)**	**N (%)**	**OR (95% CI)** ^**a**^
**Histology**			
Ductal	643,139 (72.8)	25,487 (70.2)	1.00
Lobular	73,112 (8.3)	3456 (9.5)	1.01 (0.97–1.05)
Mixed	82,546 (9.3)	3439 (9.5)	1.02 (0.98–1.06)
Others	85,084 (9.6)	3931 (10.8)	1.04 (1.00–1.08)
**Tumor grade**			
Well differentiated	165,869 (18.8)	7817 (21.5)	1.00
Moderately differentiated	337,563 (38.2)	14,526 (40.0)	0.97 (0.94–1.00)
Poorly differentiated	277,729 (31.4)	9435 (26.0)	0.91 (0.88–0.94)
Undifferentiated	9863 (1.1)	266 (0.7)	0.86 (0.76–0.98)
**Tumor size**			
0-2 cm	506,784 (57.3)	22,629 (62.3)	1.00
2-5 cm	260,786 (29.5)	9608 (26.5)	0.85 (0.83–0.87)
> 5 cm	66,236 (7.5)	1884 (5.2)	0.67 (0.64–0.71)
**Tumor stage**			
Local	547,036 (61.9)	24,624 (67.8)	1.00
Regional	265,617 (30.1)	8815 (24.3)	0.86 (0.84–0.88)
Distant	57,578 (6.5)	2065 (5.7)	0.79 (0.75–0.83)
**Molecular subtypes**^**c**^			
HR+/HER2-	205,424 (67.4)	11,106 (70.4)	1.00
HR+/HER2+	30,893 (10.1)	1283 (8.1)	0.93 (0.88–0.99)
HR−/HER2+	13,507 (4.4)	492 (3.1)	0.82 (0.75–0.90)
Triple negative	32,172 (10.6)	1481 (9.4)	1.00 (0.94–1.05)
	**N (%)**	**N (%)**	**OR (95% CI)** ^**b**^
**Surgery** ^d^			
No	60,648 (7.8)	3712 (11.6)	1.00
Lumpectomy	404,612 (52.3)	17,372 (54.1)	0.75 (0.72–0.79)
Mastectomy	304,812 (39.4)	10,900 (33.9)	0.74 (0.71–0.78)
**Chemotherapy**			
No/unknown	536,470 (60.7)	27,537 (75.8)	1.00
Yes	347,411 (39.3)	8776 (24.2)	0.84 (0.81–0.86)
**Radiotherapy**			
No/unknown	462,215 (52.3)	21,631 (59.6)	1.00
Yes	421,666 (47.7)	14,682 (40.4)	0.83 (0.82–0.85)
**Treatment modes**^**d**^			
Lumpectomy only	79,295 (10.3)	5149 (16.0)	1.00
Mastectomy only	138,766 (17.9)	6648 (20.7)	0.88 (0.85–0.92)
Chemo−/radio-therapy	27,038 (3.5)	1038 (3.2)	1.05 (0.97–1.13)
Lumpectomy plus chemo−/radio-therapy	325,317 (42.1)	12,223 (38.0)	0.79 (0.76–0.82)
Mastectomy plus chemo−/radio-therapy	166,046 (21.5)	4252 (13.2)	0.74 (0.71–0.78)
Others^e^	36,829 (4.8)	2817 (8.8)	1.16 (1.10–1.23)

NOTE. Patients with missing information on tumor stage (*N* = 14,459, 1.57%), tumor grade (*N* = 97,126, 10.56%), tumor size (*N* = 52,267, 5.68%), molecular types (*N* = 24,220, 7.6%) or surgery (*N* = 3362, 0.4%) were not included in the corresponding analysis

*Abbreviations*: *BCa-1* Breast cancer as the first malignancy, *CI* Confidence interval, *HR* Hormone-receptor, *HER2* Human epidermal growth factor receptor 2, *N* Number; *OR* Odds ratio

^a^ The models were adjusted for age (continuous) and calendar period at diagnosis, race, cohabitation status, percentile of cost of living and high-school education in county of residence

^b^ The models were additionally adjusted for tumor stage, histology, tumor grade, ER status, PR status, and HER2 status

^c^ Information on HER2 status was available from 2010 onward, and thus the analysis was restricted to patients diagnosed thereafter

^d^ Information on surgery and therapy was available from 1998 onward, and thus the analysis was restricted to patients diagnosed thereafter

^e^ Others included surgery (no/unknown), chemotherapy (no/unknown), and radiotherapy (no/unknown)

To explore the potential associations of first malignancy with BCa-2 patients, we calculated the HRs by characteristics of their first malignancies, including top ten common sites, tumor stage, and chemo−/radiotherapy for first malignancy. Because tumor characteristics and treatment modes of breast cancer might have strong impact on breast cancer specific mortality, we also performed stratified analyses and tested potential interactions between tumor characteristics, treatment modes and BCa-2 using Wald test.

All statistical analyses were conducted using STATA (version 14.1; Stata Corporation). *P* < 0.05 was considered as the statistical significance. This study was reviewed by the Biomedical Research Ethics Committee at West China Hospital, Sichuan University (reference number 2018–230).

## Results

### Demographic and clinical characteristics

Compared to patients with BCa-1, patients with BCa-2 were more likely to be diagnosed in recent years (2010–2015), older at diagnosis, and more likely to be white, not cohabitating, and residing in counties with a higher percentage of high-school education attainment (Table 1). Moreover, patients with BCa-2 had more favorable tumor characteristics (i.e., better differentiation, smaller tumor size, less advanced stage, and less likely HER2+), compared to patients with BCa-1 (Table 2). Patients with BCa-2 also received less intensive treatment, including both surgery and chemo−/radio-therapy, after controlling for differential tumor characteristics (Table 2).

Among BCa-2 patients, the most common sites of first malignancy were colon and rectum (17.8%), uterine corpus (16.7%), and blood (10.7%; Supporting Information Fig. S1). The median interval from the first malignancy to BCa-2 diagnosis was 4.75 years.

### Mortality risk in BCa-2

The median (interquartile range) of follow-up was 5.58 (2.33, 10.42) and 3.58 (1.42, 7.17) years for BCa-1 and BCa-2, respectively; and 114,964 and 3829 breast cancer-specific deaths were identified. Compared with BCa-1, BCa-2 were not associated with an increased risk of breast cancer-specific mortality (HR 1.00, 95% CI 0.97–1.03), when only controlling for demographic characteristics (Table 3). However, when accounting for tumor characteristics, we found a higher risk of breast cancer-specific mortality among patients with BCa-2 (HR 1.11, 95% CI 1.08–1.15). Additional adjustment for treatment modes led to similar association (HR 1.11, 95% CI 1.08–1.15). Given the history of a prior malignancy, patients with BCa-2 were unsurprisingly at greater increased risk of overall mortality (HR 1.56, 95% CI 1.54–1.59). Reassuringly, by restricting to BCa-2 diagnosed > 10 years after the first malignancy (i.e., presumably cured from the first malignancy and less likely misclassified for cancer-specific deaths), largely comparable association with breast cancer-specific mortality was noted (HR 1.09, 95% CI 1.02–1.16; Supporting Information Table S3). The overall mortality risk was however smaller but remained elevated compared with BCa-1 (HR 1.25, 95% CI 1.20–1.30).

**Table 3:** Hazard ratios (HRs) of cancer-specific and overall mortality among women with primary breast cancer as the second malignancy (BCa-2), compared to women with primary breast cancer as the first malignancy (BCa-1): a SEER population-based study in US, 1990–2015

	BCa-1 N (IR)	BCa-2 N (IR)	HR (95% CI)^**a**^	HR (95% CI)^**b**^	HR (95% CI)^**c**^
**All BCa-2**					
Breast cancer-specific mortality	114,964 (1.9)	3829 (2.1)	1.00 (0.97–1.03)	1.11 (1.08–1.15)	1.11 (1.08–1.15)
Overall mortality	227,860 (3.7)	13,625 (7.6)	1.46 (1.44–1.49)	1.57 (1.54–1.59)	1.56 (1.54–1.59)

*Abbreviations*: *CI* Confidence interval, *HR* Hazards ratio, *IR* Mortality rate per 100 person-years, *N* Number of deaths

^a^ HR was adjusted for age (continuous) and calendar period at diagnosis, race, cohabitation status, percentile of cost of living and high-school education in county of residence

^b^ HR was additionally adjusted for tumor stage, histology, grade, estrogen receptor status, progesterone receptor status, and human epidermal growth factor receptor 2 status

^c^ HR was additionally adjusted for surgery, radiotherapy, and chemotherapy

In a sensitivity analysis, we observed robust results after excluding childhood cancer survivors who were aged ≤20 years at the first malignancy (Supporting Information Table S4).

### Mortality risk by characteristics of the first malignancy

BCa-2 patients with a history of lung, urinary bladder, ovarian, or blood cancer were at a higher risk of breast cancer-specific mortality, whereas no risk increase was noted for patients with a previous thyroid, colorectal, skin, uterine corpus, kidney, or cervical cancer (Table 4). Moreover, stronger associations were found among BCa-2 patients whose first malignancy was at more advanced stage or treated with both chemotherapy and radiotherapy.

**Table 4:** Hazard ratios (HRs) of breast cancer-specific mortality among women with primary breast cancer as the second malignancy (BCa-2), by characteristics of the first malignancy, compared to women with primary breast cancer as the first malignancy (BCa-1): a SEER population-based study in US, 1990–2015

	N (%) of patients	N (IR) of deaths	HR (95% CI)^**a**^
**By sites of first malignancy**			
Colon and Rectum	6463 (17.8)	681 (2.1)	0.95 (0.88–1.03)
Uterine corpus	6057 (16.7)	610 (1.8)	0.98 (0.90–1.06)
Blood	3887 (10.7)	407 (2.3)	1.16 (1.05–1.28)
Skin	3489 (9.6)	309 (1.6)	0.96 (0.86–1.07)
Lung and Bronchus	3092 (8.5)	418 (4.1)	1.85 (1.68–2.03)
Thyroid	2722 (7.5)	207 (1.4)	0.93 (0.81–1.07)
Ovary	1612 (4.4)	181 (2.3)	1.19 (1.02–1.37)
Urinary Bladder	1547 (4.3)	177 (2.2)	1.22 (1.05–1.42)
Kidney	1382 (3.8)	116 (1.9)	1.01 (0.84–1.21)
Uterine cervix	1123 (3.1)	132 (2.0)	1.01 (0.85–1.20)
Others	4939 (13.6)	591 (2.9)	1.35 (1.25–1.47)
**By tumor stage of first malignancy** ^**b**^			
Localized	20,100 (62.3)	1906 (1.8)	0.98 (0.94–1.03)
Regional	7088 (22.0)	802 (2.4)	1.20 (1.12–1.29)
Distant	2661 (8.3)	379 (4.7)	1.63 (1.47–1.80)
Unstaged	2394 (7.4)	335 (3.3)	1.36 (1.22–1.51)
**By chemotherapy and radiotherapy for first malignancy**			
No/unknown	24,129 (66.4)	2580 (2.1)	1.09 (1.05–1.13)
Radiotherapy only	4816 (13.3)	470 (2.3)	1.12 (1.03–1.23)
Chemotherapy only	4796 (13.2)	505 (2.0)	1.12 (1.02–1.22)
Chemotherapy plus radiotherapy	2572 (7.1)	274 (2.9)	1.44 (1.28–1.63)

*Abbreviations*: *CI* Confidence interval, *HR* Hazards ratio, *IR* Mortality rate per 100 person-years, *N* Number of deaths

^a^ HR was adjusted for age (continuous) and calendar period at diagnosis, race, cohabitation status, percentile of cost of living and high-school education in county of residence, tumor stage, histology, tumor grade, estrogen receptor status, progesterone receptor status, and human epidermal growth factor receptor 2 status, surgery, radiotherapy, and chemotherapy

^b^ BCa-2 patients with blood malignancy as first malignancy were removed from this analysis

### Mortality risk by characteristics of breast cancer

When comparing BCa-2 to BCa-1 by the clinical characteristics of breast cancer, a stronger association with breast cancer-specific mortality was noted for patients with well-differentiated tumor or local stage, or in those only underwent lumpectomy (P for interaction< 0.05; Table 5). Similar associations were found across age or calendar year groups.

**Table 5:** Hazard ratios (HRs) of breast cancer-specific mortality among women with primary breast cancer as the second malignancy (BCa-2), stratified clinical characteristics, compared to women with primary breast cancer as the first malignancy (BCa-1): a SEER population-based study in US, 1990–2015

	BCa-1 N (IR)	BCa-2 N (IR)	HR (95% CI)^**a**^
**Age at diagnosis, years**			
18–44	19,010 (2.0)	164 (2.0)	1.05 (0.90–1.22)
45–54	25,197 (1.6)	385 (1.7)	1.05 (0.95–1.16)
55–64	25,246 (1.6)	720 (1.7)	1.19 (1.11–1.28)
65–74	20,535 (1.7)	978 (1.8)	1.15 (1.08–1.23)
75–84	16,797 (2.5)	1060 (2.5)	1.10 (1.04–1.17)
≥ 85	8179 (6.0)	522 (5.0)	1.01 (0.93–1.11)
P for interaction			0.056
**Year of diagnosis**			
1990–1993	11,532 (1.8)	344 (2.2)	1.07 (0.96–1.19)
1994–1997	13,760 (1.8)	393 (1.9)	0.98 (0.89–1.09)
1998–2001	21,259 (1.7)	516 (1.9)	1.15 (1.05–1.25)
2002–2005	26,642 (1.8)	740 (2.0)	1.15 (1.06–1.23)
2006–2009	23,333 (2.0)	890 (2.1)	1.13 (1.06–1.21)
2010–2015	18,438 (2.4)	946 (2.6)	1.14 (1.07–1.22)
P for interaction			0.136
**Histology**			
Ductal	79,321 (1.8)	2521 (2.0)	1.13 (1.09–1.18)
Lobular	9222 (1.9)	393 (2.4)	1.06 (0.95–1.17)
Mixed	8133 (1.4)	314 (1.8)	1.20 (1.08–1.35)
Others	18,288 (3.2)	601 (3.5)	1.03 (0.95–1.12)
P for interaction			0.067
**Tumor grade**			
Well differentiated	5445 (0.5)	294 (0.7)	1.43 (1.27–1.61)
Moderately differentiated	31,639 (1.4)	1149 (1.6)	1.15 (1.09–1.22)
Poorly differentiated	53,944 (3.0)	1593 (3.6)	1.18 (1.12–1.24)
Undifferentiated	2400 (2.9)	52 (3.1)	1.07 (0.82–1.41)
P for interaction			0.011
**Tumor size**			
0–2 cm	28,594 (0.7)	1264 (1.0)	1.29 (1.22–1.36)
2–5 cm	45,123 (2.7)	1434 (3.5)	1.15 (1.09–1.21)
> 5 cm	23,418 (7.4)	563 (8.6)	1.25 (1.15–1.36)
P for interaction			0.011
**Tumor stage**			
Local	27,833 (0.7)	1293 (1.0)	1.31 (1.24–1.39)
Regional	48,765 (2.7)	1398 (3.3)	1.07 (1.01–1.13)
Distant	33,806 (21.2)	981 (26.1)	1.03 (0.97–1.10)
P for interaction			< 0.001
**Molecular subtypes**^b^			
HR+/HER2-	8076 (1.5)	447 (1.7)	1.09 (0.99–1.20)
HR+/HER2+	1743 (2.3)	75 (2.6)	1.38 (1.09–1.74)
HR−/HER2+	1281 (3.9)	62 (5.5)	1.61 (1.25–2.08)
Triple negative	4322 (5.5)	213 (6.7)	1.18 (1.03–1.36)
P for interaction			0.020
**Treatment modes**^c^			
Lumpectomy only	5816 (1.2)	349 (1.7)	1.32 (1.19–1.47)
Mastectomy only	11,190 (1.3)	510 (1.6)	1.13 (1.03–1.23)
Chemo−/radio-therapy	12,602 (16.5)	365 (17.9)	1.14 (1.03–1.27)
Lumpectomy plus chemo/radio-therapy	17,627 (0.8)	555 (0.9)	1.13 (1.03–1.23)
Mastectomy plus chemo/radio-therapy	28,121 (2.8)	649 (3.3)	1.13 (1.04–1.22)
Others^d^	14,316 (13.9)	664 (14.1)	0.99 (0.92–1.07)
P for interaction			0.002

NOTE. Patients with missing information on tumor stage (*N* = 14,459, 1.57%), tumor grade (*N* = 97,126, 10.56%) or size (*N* = 52,267, 5.68%) or molecular types (*N* = 24,220, 7.6%) were not included for the corresponding analysis. We added an interaction term between BCa-2 and the risk modifier and reported the statistical significance of the term as P for interaction

*Abbreviations*: *CI* Confidence interval, *HR+* Hormone-receptor positive, *HR*- Hormone-receptor negative, *HER2* Human epidermal growth factor receptor 2, *HR* Hazard ratio, *IR* Mortality rate per 100 person-years, *N* Number of deaths

^a^ HR was adjusted for age (continuous) and calendar period at diagnosis, race, cohabitation status, percentile of cost of living and high-school education in county of residence, tumor stage, histology, tumor grade, estrogen receptor status, progesterone receptor status, and human epidermal growth factor receptor 2 status, surgery, radio-therapy, and chemo-therapy

^b^ Information on HER2 status was available from 2010 onward, and thus the analysis was restricted to patients diagnosed thereafter

^c^ Information on surgery was available from 1998 onward, and thus the analysis was restricted to patients diagnosed thereafter

^d^ Others included surgery (no/unknown), chemotherapy (no/unknown), and radiotherapy (no/unknown)

## Discussion

To the best of our knowledge, this is the first study to assess the mortality risk for BCa-2 as one entity among all cancer survivors. In this population-based cohort study, we found that, although with more favorable tumor characteristics and receiving less intensive treatment, BCa-2 were independently associated with an increased risk of breast cancer-specific mortality, compared with BCa-1. The risk increase was particularly greater when the first malignancy was lung, urinary bladder, ovarian, or blood malignancy, at more advanced stage, or treated with both chemotherapy and radiotherapy. Stronger associations were also found for BCa-2 with favorable characteristics or only treated by lumpectomy.

Interestingly, the breast-cancer specific mortality was comparable among patients with BCa-2 and BCa-1 when only accounting for demographic characteristics, likely explained by a mixed effect of poorer prognosis but more favorable tumor features (e.g., less advanced stage) in BCa-2. However, the inferior prognosis of BCa-2 is evident when controlling for tumor characteristics and treatment modes. Indeed, we lacked detailed information on regimens of chemo−/radio-therapy, and therefore were not able to eliminate the residual effect, if any, of differential regimens on BCa-2 prognosis. However, BCa-2 patients received less intensive treatment than their BCa-1 counterparts given the same demographic (e.g., age at diagnosis) and tumor characteristics (e.g., tumor stage and molecular subtype).

Previous studies suggested that BCa-2 subsequent to HL were associated with a higher risk of breast cancer-specific mortality, compared with BCa-1 [15, 18]. In the present study, we also observed worse prognosis in BCa-2 among patients with hematopoietic and lymphoid malignancies, which accounted for 10% of all BCa-2 cases. Importantly, we extend the current knowledge to BCa-2 among other cancer survivors, especially in survivors of lung, ovarian, or bladder cancers. It is conceivable that the worsened mortality among these patients may stem from mutations shared between BCa-2 and other malignancies or undertreatment, if any, of BCa-2 in the face of a competing, presumably the more life-threatening first malignancy. Indeed, BCa-2 with a history of many less fatal cancer types are not at increased risk of breast cancer-specific mortality. Prior cancer is a common exclusion criterion in clinical trials due to concerns that prior cancer may affect trial conduct or outcomes [26]. Our findings therefore may add to the ongoing discussion in support of the broader inclusion of BCa-2 subgroups that were not of poorer prognosis in clinical trials. Moreover, a recent report suggested childhood cancer survivors (age at first primary malignancy ≤20 years) with BCa-2 tended to have an modestly increased, although not statistically significant, risk of breast cancer-specific mortality than individuals with BCa-1 (HR 1.3, 95% CI 0.9–2.0) [27]. In our sensitivity analysis, the increased risk remained the same after excluding childhood cancer survivors. We, therefore, add to the knowledge by revealing the worse prognosis of BCa-2 developed in adulthood cancer survivors.

The major strength of our study is the large-scale population-based prospective cohort of patients with primary breast cancer, which assures minimal biases including selection and surveillance biases. One of the major concerns is that some deaths due to the first malignancy were misclassified as deaths due to breast cancer, which may lead to overestimated associations. However, we performed a sensitivity analysis by restricting to BCa-2 diagnosed > 10 years, i.e., presumably cured, from the first malignancy which yielded similar association with breast cancer-specific mortality. Our findings are therefore unlikely explained by the misclassification (if any) of deaths due to first malignancy. Second, we lacked information on a few factors that may be associated with survival, e.g., performance status [28], body mass index [29] and comorbidities [30], which may differ between individuals with multiple cancers and patients with BCa-1. However, these factors are likely influential through treatment modes which have been carefully addressed in our analyses. Third, the ascertainment of some BCa-2 may be challenged if the first malignancy was metastatic. However, as pathological diagnosis is required for inclusion, it is unlikely that the identified BCa-2 are in fact metastasis from the first primary malignancy. Also, breast metastasis from non-mammary malignancies is rare, accounting for approximately 1.8% of all breast malignancies [31]. In addition, we have shown the increased breast cancer-specific mortality for BCa-2 patients with regional stage of first malignancy. Lastly, the follow-up in our study is relatively short. Future studies with longer follow-up are needed.

Several potential mechanisms may help explain the unfavorable prognosis of BCa-2. First, it is possible that BCa-2 are different from BCa-1 in tumor biology. Similar to a previous study on BCa-2 after HL [15], our data indicate that BCa-2, in general, are characterized by less aggressive tumor at diagnosis, including early stage and smaller size, potentially due to the early detection during the clinical follow-up for the first malignancy. However, as our data suggest that the non-aggressive clinical features did not translate to a better prognosis of BCa-2. Behrens et al. reported [32] the overall frequency of microsatellite alterations in BCa-2 after HL was substantially higher than that in BCa-1, potentially influenced by immunosuppression and radiation exposure due to HL. A line of research also suggests that ionizing radiation may lead to breast carcinogenesis via radiation-induced amplification of proto-oncogene c-MYC [33]. Synergistic effect on a second malignancy has been postulated when the first malignancy was treated with both radiation and chemotherapy [34]. Interestingly, we found greater risk increase of breast cancer-specific mortality in BCa-2 patients who underwent chemotherapy and radiotherapy for the first malignancy. Moreover, genetic susceptibility may contribute to the multiple malignancies in an individual. For example, genetic variants in BRCA locus are associated with breast and ovarian cancers [11]. It is plausible that BCa-2 developed in survivors of ovarian cancer are driven by shared genetic variants and, thus, different from sporadic cases. This may partly explain our findings on the particularly worse prognosis of BCa-2 after ovarian cancer. A recent study also showed that breast cancer developed between two screenings, a known type of worse prognosis, is more likely to have a non-breast malignancy before and after breast cancer diagnosis, potentially through rare deleterious mutations in cancer genes [12]. Further research is warranted to understand mutations leading to BCa-2 after other malignancies.

Second, the clinical management for BCa-2 may be less intensive than BCa-1. This has been acknowledged in previous studies, suggesting that BCa-2 in HL survivors were less treated than BCa-1 with similar tumor characteristics [15, 17]. The present study further confirmed that BCa-2 in cancer survivors were in general less treated, particularly when the first malignancy is considered of poor prognosis. For instance, we found greater risk increase of breast cancer-specific mortality in BCa-2 when the first malignancy was diagnosed at a more advanced stage, even though we exhaustively controlled for treatment modes.

## Conclusions

Our findings suggest that, overall, patients with BCa-2 have worse breast cancer-specific survival, compared with their BCa-1 counterparts, although the risk elevation is mild. Active clinical management may be considered for high-risk groups, for example, patients with a prior lung, bladder, ovarian, or blood malignancy. On the other hand, our findings may add to the current discussion in support of broader inclusion of the other BCa-2 subgroups, who have a comparable prognosis with BCa-1, in clinical trials. Future research is also required to understand the potential difference between BCa-2 and BCa-1 regarding tumor biology.

## Supplementary Information

**Additional file 1: Table S1.** Baseline characteristics and mortality of women indicated as BCa-2 but no information on the first malignancy in the SEER. **Table S2.** Site recode definitions of first primary malignancies among patients with second primary breast cancer. **Table S3.** Hazard ratios (HRs) of breast cancer-specific and overall mortality among women with primary breast cancer as the second malignancy (BCa-2) diagnosed > 10 years after first malignancy, compared to women with primary breast cancer as the first malignancy (BCa-1): a SEER population-based study in US, 1990–2015. **Table S4.** Hazard ratios (HRs) of breast cancer-specific and overall mortality among adulthood cancer survivors with primary breast cancer as the second malignancy (BCa-2), compared to women with primary breast cancer as the first malignancy (BCa-1): a SEER population-based study in US, 1990–2015. Childhood cancer survivors who were < 20 years old at first primary malignancy (*n* = 192) were excluded in this analysis. **Fig. S1.** Ten most common sites of first malignancy among women with primary breast cancer as the second malignancy: a SEER population-based study in US, 1990–2015.

## Data Availability

The data is publicly available from the SEER Program (https://seer.cancer.gov/).

## Abbreviations

BCa-2: Cancer survivors who developed breast cancer as a second malignancy
BCa-1: Patients with breast cancer as the first malignancy
HR: Hazard ratio
CBC: Contralateral breast cancer
HL: Hodgkin’s lymphoma
SEER: Surveillance, Epidemiology, and End Results
ER: Status of estrogen receptor
PR: Progesterone receptor
HER2: Human epidermal growth factor receptor 2
HR+: Hormone receptor positive
HR: Hormone receptor negative
