# Supplementary material for: Increased risk of breast cancer-specific mortality among cancer survivors who developed breast cancer as a second malignancy
Source: BMC Cancer. 2021 May 3;21:491. doi: 10.1186/s12885-021-08132-9 (PMC8091680; doi:10.1186/s12885-021-08132-9)
Supplement: Supplementary file 1 — Additional file 1: Table S1. Baseline characteristics and mortality of women indicated as BCa-2 but no information on the first malignancy in the SEER. Table S2. Site recode definitions of first primary malignancies among patients with second primary breast cancer. Table S3. Hazard ratios (HRs) of breast cancer-specific and overall mortality among women with primary breast cancer as the second malignancy (BCa-2) diagnosed > 10 years after first malignancy, compared to women with primary breast cancer as the first malignancy (BCa-1): a SEER population-based study in US, 1990–2015. Table S4. Hazard ratios (HRs) of breast cancer-specific and overall mortality among adulthood cancer survivors with primary breast cancer as the second malignancy (BCa-2), compared to women with primary breast cancer as the first malignancy (BCa-1): a SEER population-based study in US, 1990–2015. Childhood cancer survivors who were < 20 years old at first primary malignancy (n = 192) were excluded in this analysis. Fig. S1. Ten most common sites of first malignancy among women with primary breast cancer as the second malignancy: a SEER population-based study in US, 1990–2015. [file 12885_2021_8132_MOESM1_ESM.docx]

| **Table S1. Baseline characteristics and mortality of women indicated as BCa-2 but no information on the first malignancy in the SEER.** | | |
| --- | --- | --- |
|  | **N** | **%** |
| **Total number** | 41,572 | - |
| **Year of diagnosis** |  |  |
| 1990-1993 | 2,047 | 4.9 |
| 1994-1997 | 2,778 | 6.7 |
| 1998-2001 | 6,515 | 15.7 |
| 2002-2005 | 8,916 | 21.4 |
| 2006-2009 | 8,642 | 20.8 |
| 2010-2015 | 12,674 | 30.5 |
| **Age at diagnosis (mean±SD), years** | 67.7**±**13.4 | |
| **Race** |  |  |
| White | 35,864 | 86.3 |
| Black | 3,921 | 9.4 |
| Asian | 1,562 | 3.8 |
| Other | 225 | 0.5 |
| **Cohabitation status** |  |  |
| Non-cohabitation | 20,086 | 48.3 |
| Cohabitation | 19,539 | 47.0 |
| Unknown | 1,947 | 4.7 |
| **% of High-school education in the county of residence** |  |  |
| Lowest tertile | 8,829 | 21.2 |
| Middle tertile | 16,952 | 40.8 |
| Highest tertile | 15,791 | 38.0 |
| **Cost of living adjusted median household income in the county of residence** |  |  |
| Lowest tertile | 3,118 | 7.5 |
| Middle tertile | 6,540 | 15.7 |
| Highest tertile | 31,914 | 76.8 |
| **Histology** |  |  |
| Ductal | 29,111 | 70.0 |
| Lobular | 3,834 | 9.2 |
| Mixed | 4,166 | 10.0 |
| Others | 4,461 | 10.7 |
| **Tumor grade** |  |  |
| Well differentiated | 8,213 | 19.8 |
| Moderately differentiated | 16,330 | 39.3 |
| Poorly differentiated | 11,644 | 28.0 |
| Undifferentiated | 551 | 1.3 |
| Unknown | 4,834 | 11.6 |
| **Tumor size** |  |  |
| 0-2cm | 25,703 | 61.8 |
| 2-5cm | 10,894 | 26.2 |
| >5cm | 2,423 | 5.8 |
| Unknown | 2,552 | 6.1 |
| **Tumor stage** |  |  |
| Local | 27,716 | 66.7 |
| Regional | 10,589 | 25.5 |
| Distant | 2,564 | 6.2 |
| Unknown | 703 | 1.7 |
| **Molecular types^a^** |  |  |
| HER2+/HR+ | 1,041 | 8.2 |
| HER2+/HR- | 436 | 3.4 |
| HER2-/HR+ | 8,760 | 69.1 |
| Triple negative | 1,315 | 10.4 |
| Unknown | 1,122 | 8.9 |
| **Treatment modes^b^** |  |  |
| Surgery only | 16,129 | 43.9 |
| Surgery plus chemo-/radio-therapy | 17,362 | 47.2 |
| chemo-/radio-therapy | 1,042 | 2.8 |
| Others^c^ | 2,214 | 6.0 |
|  | **N of deaths** | **IR** |
| **Any cancer-specific mortality** | 7,296 | 2.8 |
| Breast cancer-specific mortality | 5,892 | 2.3 |
| Other cancer-specific mortality | 1,404 | 0.5 |
| **Overall mortality** | 15,532 | 6.0 |

Abbreviations: HR, hormone-receptor; HER2, human epidermal growth factor receptor 2; IR, mortality rate per 100 person-years; N, number; SD, standard deviation.

^a^ Information on HER2 status was available from 2010 onward, and thus the analysis was restricted to patients diagnosed thereafter.

^b^ Information on surgery was available from 1998 onward, and thus the analysis was restricted to patients diagnosed thereafter.

^c^ Others included surgery (no/unknown), chemotherapy (no/unknown), and radiotherapy (no/unknown).

**Table S2. Site recode definitions of first primary malignancies among patients with second primary breast cancer.**

| **Classification of first primary malignancies** | **Site recodes in SEER** |
| --- | --- |
| Colon and rectum | 21041-21052 |
| Corpus and uterus | 27020, 27030 |
| Blood | 33041-35043 |
| Skin | 25010, 25020 |
| Lung and bronchus | 22030 |
| Thyroid | 32010 |
| Ovary | 27040 |
| Urinary bladder | 29010 |
| Kidney | 29020 |
| Cervix uteri | 27010 |
| Others | The remaining codes |

**Table S3. Hazard ratios (HRs) of breast cancer-specific and overall mortality among women with primary breast cancer as the second malignancy (BCa-2) diagnosed >10 years after first malignancy, compared to women with primary breast cancer as the first malignancy (BCa-1): a SEER population-based study in US, 1990-2015.**

|  | **BCa-1**  **N (IR)** | **BCa-2**  **N (IR)** | **HR (95% CI)^a^** | **HR (95% CI)^b^** | **HR (95% CI)^c^** |
| --- | --- | --- | --- | --- | --- |
| **BCa-2 diagnosed >10 years after first malignancy** | | | | | |
| Breast cancer-specific mortality | 114,964 (1.9) | 943 (2.0) | 0.98 (0.92-1.04) | 1.07 (1.01-1.14) | 1.09 (1.02-1.16) |
| Overall mortality | 227,860 (3.7) | 2,917 (6.3) | 1.17 (1.12-1.21) | 1.25 (1.20-1.29) | 1.25 (1.20-1.30) |

Abbreviations: CI, confidence interval; HR, hazards ratio; IR, mortality rate per 100 person-years; N, number of deaths.

^a^ HR was adjusted for age (continuous) and calendar period at diagnosis, race, cohabitation status, percentile of cost of living and high-school education in county of residence.

^b^ HR was additionally adjusted for tumor stage, histology, grade, estrogen receptor status, progesterone receptor status, and human epidermal growth factor receptor 2 status.

^c^ HR was additionally adjusted for surgery, radiotherapy, and chemotherapy.

| **Table S4. Hazard ratios (HRs) of breast cancer-specific and overall mortality among adulthood cancer survivors with primary breast cancer as the second malignancy (BCa-2), compared to women with primary breast cancer as the first malignancy (BCa-1): a SEER population-based study in US, 1990-2015.** Childhood cancer survivors who were <20 years old at first primary malignancy (n=192) were excluded in this analysis. | | | | |
| --- | --- | --- | --- | --- |
|  | **BCa-2**  **N (IR)** | **HR (95%CI)^a^** | **HR (95%CI)^b^** | **HR (95%CI)^c^** |
| Breast cancer-specific mortality | 3,797 (2.1) | 1.00 (0.96-1.03) | 1.11 (1.07-1.15) | 1.11 (1.07-1.15) |
| Overall mortality | 13,574 (7.6) | 1.46 (1.44-1.49) | 1.56 (1.54-1.59) | 1.56 (1.53-1.59) |
| Abbreviations: CI, confidence interval; HR, hazards ratio; IR, mortality rate per 100 person-years; N, number of deaths.  ^a^ HR was adjusted for age (continuous) and calendar period at diagnosis, race, cohabitation status, percentile of cost of living and high-school education in county of residence.  ^b^ HR was additionally adjusted for tumor stage, histology, grade, estrogen receptor status, progesterone receptor status, and human epidermal growth factor receptor 2 status.  ^c^ HR was additionally adjusted for surgery, radiotherapy, and chemotherapy. | | | | |

**Fig S1.** **Ten most common sites of first malignancy among women with primary breast cancer as the second malignancy: a SEER population-based study in US, 1990-2015.**

**
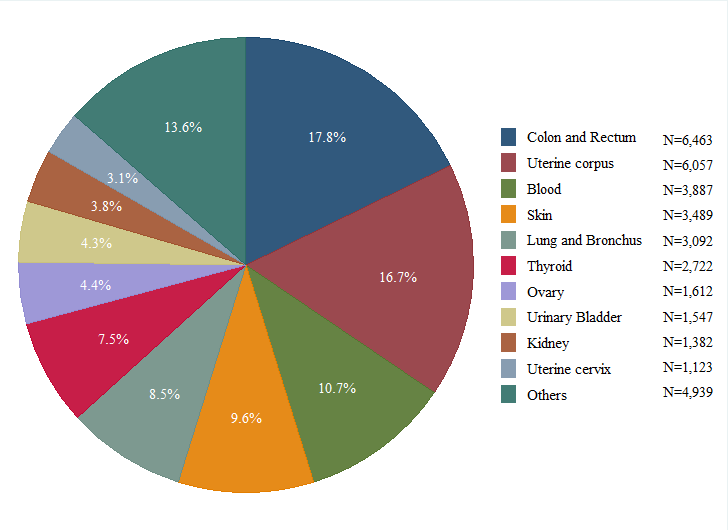
**
